# Evolution of Genetic Techniques: Past, Present, and Beyond

**DOI:** 10.1155/2015/461524

**Published:** 2015-03-22

**Authors:** Asude Alpman Durmaz, Emin Karaca, Urszula Demkow, Gokce Toruner, Jacqueline Schoumans, Ozgur Cogulu

**Affiliations:** ^1^Department of Medical Genetics, Ege University Faculty of Medicine, 35100 Izmir, Turkey; ^2^Department of Laboratory Diagnostics and Clinical Immunology, Warsaw University Faculty of Medicine, 61 02-091 Warsaw, Poland; ^3^Institute of Genomic Medicine, UMDNJ-NJ Medical School, Newark, NJ 07103, USA; ^4^Department of Medical Genetics, Cancer Cytogenetic Unit, Lausanne University Hospital, 1011 Lausanne, Switzerland

## Abstract

Genetics is the study of heredity, which means the study of genes and factors related to all aspects of genes. The scientific history of genetics began with the works of Gregor Mendel in the mid-19th century. Prior to Mendel, genetics was primarily theoretical whilst, after Mendel, the science of genetics was broadened to include experimental genetics. Developments in all fields of genetics and genetic technology in the first half of the 20th century provided a basis for the later developments. In the second half of the 20th century, the molecular background of genetics has become more understandable. Rapid technological advancements, followed by the completion of Human Genome Project, have contributed a great deal to the knowledge of genetic factors and their impact on human life and diseases. Currently, more than 1800 disease genes have been identified, more than 2000 genetic tests have become available, and in conjunction with this at least 350 biotechnology-based products have been released onto the market. Novel technologies, particularly next generation sequencing, have dramatically accelerated the pace of biological research, while at the same time increasing expectations. In this paper, a brief summary of genetic history with short explanations of most popular genetic techniques is given.

## 1. Introduction

**Figure 1:**
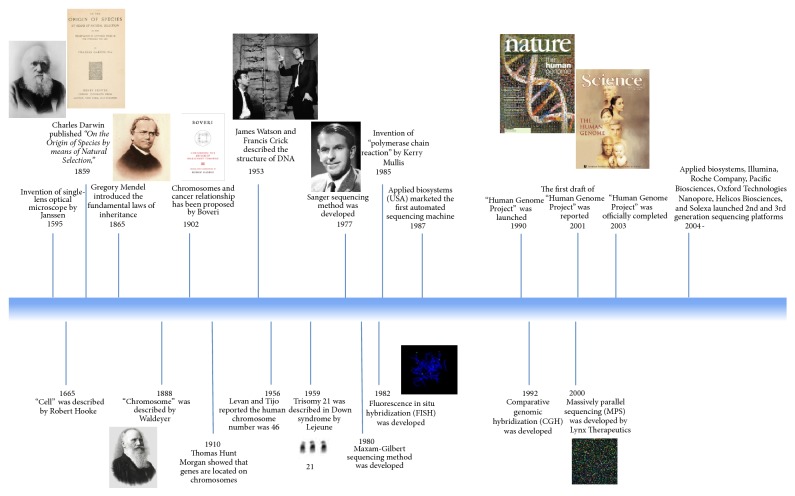
Landmarks in the history of genetics.

Due to rapid advances in genomic technologies, genetics analyses have become essential in clinical practice and research. During the past decade, a great stride has been made to unravel underlying mechanisms of genetic-related disorders. Landmarks in genetic history are summarized in Figure 1. Moreover, genetic testing methods have become widely accessible and feasible to perform even for small size laboratories in particular after the completion of Human Genome Project, which coincided with developments in computer technology. With the application of genetic testing for personalized medicine, we are at the beginning of an era that will provide new horizons in human health.

## 2. History of Genetic Techniques and Properties of Methods

### 2.1. Conventional Cytogenetic Techniques

Looking at the history in brief, genetics is the term introduced for the study of genes in organisms. Many early discoveries contributed as important milestones to evolve the study of genomes as it is applied today. One of the crucial steps that enabled visualizing intracellular structures was the invention of the single-lens optical microscope by Janssen in 1595 [1]. After many researchers started to make observations with the newly invented microscope, Hooke has proposed the description of the “cell” in 1665 [2]. A Swiss botanist Nageli first described thread-like structures in the nuclei of plant cells in 1840s, what he called “transitory cytoblasts” which would be defined as chromosomes later on by Waldeyer in 1888 [3]. After Charles Darwin published* On the Origin of Species by means of Natural Selection* in 1859, Gregor Mendel introduced the fundamental laws of inheritance in 1865. This was crucial for understanding that “some characteristics of the organisms are inherited through genes.” Mendel's rules were improved by the experiments of Thomas Hunt Morgan in 1910 who discovered that genes were responsible for the appearance of a specific phenotype located on chromosomes. In 1911, the first genetic map was achieved by mapping the fruit fly genes. From the 1800s until the middle of the 1900s, it was not understood that the structure of hereditary genetic material was responsible for the inheritance of traits from one generation to the next. In 1953, Watson and Crick described the double-stranded, helical, complementary, and antiparallel model for DNA [4]. They shared Nobel Prize in Medicine for this major discovery of the structure of DNA in 1962. In 1966, the genetic code in the DNA was finally discovered by defining that a codon which is a sequence of adjacent 3 nucleotides codes for the amino acids. The discovery of DNA and chromosomes paved the way for the rapid improvement in genetics and establishment of new technologies that have taken place over the last 50 years.

The first genetic analysis was performed in the field of cytogenetics. Although it was published that normal human chromosome number was 48, Tjio and Levan reported in 1956 that the correct number was 46 [5]. After the establishment of the peripheral leucocyte culture method incorporation with the fixation and staining methods, it became possible to identify human chromosome abnormalities associated with specific congenital defects [6]. Just a few years later, when Tjio and Levan reported the correct human chromosome number, several reports identifying numerical chromosome abnormalities such as trisomy 21 in Down syndrome, monosomy X and XXY in two frequent sex chromosomal disorders, Turner and Klinefelter syndromes, respectively, were published in 1959 [7–9]. After 1966, genetic techniques were performed not only in the postnatal samples but also in the prenatal samples, as it was shown that fetal cells derived from amniotic fluid could be obtained by using an invasive procedure which is termed as amniocentesis. Steele and Breg reported that cells cultured from amniotic fluid could be used to determine the chromosome aberrations in the fetus [10]. However, the resolution of the chromosomes was not high enough to determine the structural aberrations in cultured cells which was improved by the high resolution banding techniques using synchronized lymphocyte cultures established by Yunis in 1976 [11]. This novel banding technique allowed identifying the genetic etiology of clinically well-known syndromes such as Cri-du-Chat and Wolf-Hirschhorn syndromes. Besides unbalanced aberrations in patients, underlying chromosomal anomalies in those cases having balanced translocations with a history of recurrent miscarriages or having a deceased child with multiple congenital anomalies, have also been described by the use of the high resolution banding techniques. The relation between chromosomes and cancer was also established by Boveri in 1902. According to Boveri's somatic mutation theory, cancer is caused by at least one mutation in the cells which causes defect in control of the cell proliferation and division [12]. He emphasized that the underlying main reason was abnormal chromosomal changes in a cancer cell.

### 2.2. Molecular Cytogenetic Techniques

Despite the establishment of high-resolution techniques which enabled revealing many known or unknown genetic syndromes, several cases having submicroscopic aberrations that were not visible at resolution between 500 and 1000 bands remained undiagnosed. A new technique called fluorescence in situ hybridization (FISH) was developed in the field of molecular cytogenetics in 1982 [13]. In this method, cytogenetics and molecular genetics were bridged. FISH identifies specific nucleic acid sequences from interphase nuclei or applied on metaphase chromosomes of [14, 15]. While this technique has advanced significantly today, it was previously based on the radioactively labeled ribosomal RNA hybridized to acrocentric chromosomes followed-up visualizing hybridization by autoradiography. Several techniques before fluorescence based techniques such as enzyme-based and gold-based probe systems were also used in the past [16]. The first application of FISH was established in 1980, when RNA that was directly labeled with fluorophore was used as a probe for specific DNA sequences [17]. Langer et al. developed a new technique involving the use of a nonradioactive probe for indirect labeling through nick translation [18]. In later times, by the development of new fluorescent molecules, which led to direct and indirect fluorescent labeled probe, binding to DNA bases improved the protocols of FISH. Chromosome rearrangements could be detected more easily with increased resolution of the FISH in both metaphases and interphases nuclei that could be used for both clinical diagnosis and research. FISH also provided the option for the simultaneous use of one or more DNA probes by labeling different colors or color combinations. Several types of probes can be used for FISH. Whole-chromosome painting probes, chromosome-arm painting probes, and centromeric, subtelomeric, and locus-specific probes are some of the examples which are available for the detection of specific constitutional and acquired chromosomal abnormalities. Consequently, a large number of sophisticated approaches were established based on the FISH-methods, for example, SKY (spectral karyotyping FISH) [19], Q-FISH (quantitative FISH) [20], fiber-FISH [21], heterochromatin-M-FISH [22] (M-FISH multicolor FISH) [23], COBRA-FISH (combined binary ratio labeling FISH) [24], cenM-FISH (centromere-specific M-FISH) [25], and other modified FISH approaches. The most advanced FISH-based approaches for whole-chromosome analysis are COBRA-FISH, M-FISH, and SKY FISH. Hybridizing all 24 different human chromosomes with whole-chromosome painting probes labeled with a combination of 5 different fluorophores enables visualizing each chromosome with a specific color. FISH also enabled showing that chromosomes are compartmentalized into discrete territories in the nucleus [26]. A correlation between the location and the size and the gene content of the chromosomes was described. Smaller and gene-rich chromosomes are generally situated towards the interior whereas larger and gene-poor chromosomes are generally situated towards the periphery of the nucleus [27, 28]. With the accurate map of the human genome obtained by the Human Genome Project, more and more probes from cloned and mapped segments (cosmids, PACs, BACs, and YACs) have become available for diagnostic purposes [29]. In light of these studies, several clinical diagnostic FISH tests have become commercially available. The most remarkable one is the test for deletion or duplication of the subtelomeric regions leading to a clinical picture mostly characterized by multiple congenital anomalies and intellectual disability. Subtelomeric aberrations are found in approximately 5% of patients [30–32] and this finding suggested that submicroscopic aberrations (i.e., deletions and duplications) might be present across the genome, particularly in patients demonstrating similar phenotypes with normal cytogenetic investigations.

Being time-consuming and expensive to evaluate chromosomal rearrangements in the whole genome by FISH led to the development of new techniques such as array-based comparative genomic hybridization [33–35]. Comparative genomic hybridization (CGH) which is based on competitive hybridization of amplified tumor DNA and normal DNA hybridized on normal metaphase slide was first developed by Kallioniemi et al. in 1992 to detect genomic imbalances in tumor cells [36]. If we look at the evolution of genetic technologies, the emergence of new technologies has always been inevitable due to the necessities revealed by the previous technologies. Although hybridization-based methods, which allow screening RNA, DNA, or protein such as northern blot, southern blot, or western blot, respectively, are widely used, innovative and powerful microarray hybridization methods were developed in the 1990s. Instead of hybridizing a labeled probe to targeted DNA on a slide, with array-CGH, the patient's DNA is hybridized to a large number of well-characterized probes immobilized on a slide. To summarize briefly, in this method, DNA of the patient is labeled with a specific color (green) and mixed with exactly the same amount of DNA of a normal control, which is labeled with a different color (red). This DNA mixture is then hybridized to the denaturated probe DNA on the glass and signal intensity ratios of test over reference are measured. Yellow dye appears when both patient and reference DNA are equal in proportion because of the presence of the same amount of red and green dyes, while regions with copy number losses are visualized as red and gains are green. This technique permits the detection of whole genome copy number variation (CNV) (duplications and deletions) at high resolution [36].

Array-CGH failed to detect the recessive disease genes, mosaic aneuploidy, uniparental disomy (UPD), or heterochromatic rearrangements. Because of this disadvantage, it is thought to combine with SNP arrays to improve the resolution of array-CGH [37]. These arrays have the highest resolution of all of the available array-based platforms. They have 10–15 times higher resolution when compared with FISH analysis (5–10 kb) [38]. Combination of array-CGH and SNP genotyping in a single platform increases the clinical diagnostic capability and uncovers the detection of small copy number variants [39]. In addition, array-based CGH has the advantage over FISH in the fact that living cells are not needed to obtain metaphase chromosomes because only DNA is needed for analysis. The major drawback of array-based CGH is that it can only detect unbalanced rearrangements and is unable to detect balanced aberrations such as chromosome translocations, inversions, and insertions. However, recently, a modified array protocol, called translocation CGH (tCGH), was developed to address recurrent translocation breakpoints [40].

### 2.3. Molecular Genetics

Larger genomic changes such as deletions, duplications, and translocations can be detected by conventional karyotyping, FISH, or array-CGH methods but single nucleotide changes cannot be detected by these techniques. Molecular genetic techniques were rapidly developed after the establishment of polymerase chain reactions that enabled generating thousands to millions of copies of a particular DNA sequence [41]. Mullis and Smith have shared the 1993 Nobel Prize in Chemistry for their discovery of the polymerase chain reaction (PCR) technique. Although PCR was developed only a few decades ago, it has found numerous basic and clinical applications and is indispensable in today's science.

Taq DNA polymerase that was selected the first “Molecule of the Year” by Journal of Science was a major advantage in PCR technology [42]. Automation of PCR was greatly facilitated and simplified the detection of genomic mutations. PCR was previously used by the following techniques such as restriction fragment length polymorphism (RFLP), single-strand confirmation polymorphism (SSCP), and sequencing based methods. SSCP and RFLP, the most widely used techniques for mutation screening method in genetic diagnostic laboratories, were not able to detect every mutation, so development of new methods was needed. If the sequence of the gene of interest is not known, it may be difficult to interpret the results of these techniques. The determination of DNA sequencing enabled identifying the definite nucleotide changes in the targeted genes. This necessity was overcome by Maxam and Gilbert introducing Maxam-Gilbert chemical sequencing technology based on chemical modification of DNA followed by cleavage at specific bases [43]. Despite the efficiency of Maxam-Gilbert sequencing method, the use of hazardous chemical and inability to read long PCR fragments made this method replaced by Sanger sequencing that was based on dideoxynucleotide chain termination [44]. Manual Sanger sequencing method has been improved by the introduction of first generation of automated DNA sequencers [45]. Automatization of DNA sequencing enabled sequencing human genome in a fast and accurate way.

With the advances in the field of molecular genetics, it became possible to launch the Human Genome Project to reveal the complete human genome. The programme was launched in the USA with an effort of the Department of Energy and the National Institutes of Health in collaboration involving 20 groups in 1990. The first draft of human genome was published in 2001 by The International Human Genome Sequencing Consortium [46]. This first report covering 94% of the human genome announced that the human genome had 30,000–40,000 protein-coding genes and more than 1.4 million single nucleotide polymorphisms (SNPs). One day later, in parallel with HGP, Craig Venter who launched a human genome sequencing project by Celera Genomics using shot-gun sequencing method published the whole human genome sequence in Science [47]. The project was declared to be finished two and a half years ahead of scheduled time in 2003 coinciding with the 50th anniversary of the paper in which Watson and Crick reported DNA's double helix [48]. It was reported that 20,000–25,000 genes were present in the human genome covering 93% of the euchromatic region. Human Genome Project not only revealed the complete sequence of the human genome but also led to a huge improvement in the sequencing technology. Amplification of the gene of interest in the affected individual(s) enabled revealing mutations associated with specific monogenic disorders. Although automation of traditional dideoxy DNA sequencing Sanger method increases the efficiency of DNA sequencing, it was still not cost- and time-effective. A new technology called massively parallel sequencing (MPS) erasing these disadvantages was developed by Lynx Therapeutics [49]. This technology using reads of multiple reactions simultaneously and generating large amounts of sequence data in parallel provided a large impetus for exome sequencing, whole genome sequencing, and transcriptome and methylation profiling. This high-throughput technology that is called next generation sequencing (NGS) technology reduced the cost of sequencing of a human genome to less than $1.000. This technology is projected to sequence a human genome in 1 hour for $100 after new technological improvements in the near future. NGS technology is widely used for a variety of clinical and research applications, such as detection of rare genomic variants by whole genome resequencing or targeted sequencing, transcriptome profiling of cells, tissues, and organisms, and identification of epigenetic markers for disease diagnosis. One of the most successful applications of NGS technology is genome-wide discovery of causal variants in single gene disorders and complex genomic landscapes of many diseases. While whole genome or whole exome sequencing is the most comprehensive strategy in the diagnosis of unknown diseases and identification of new disease genes, targeted sequencing using selected panels of genes can reduce the sequencing time and cost by combining the diseases in the same group or pathway genes in known clinical pictures such as intellectual disability, neurometabolic disorders, or malignancies [50].

In addition to cost-effective advantages, sequencing the small part of the genome allows reducing the number of variations that in turn reduce the cost and time needed for data interpretation [51]. Targeted sequencing opened a new window in the diagnosis of several diseases with unknown etiology. For instance, it is possible to analyze more than 4800 genes which were identified in the Human Gene Mutation Database (HGMD Professional) and the Online Mendelian Inheritance in Man (OMIM) catalog in a single run for patients having unknown genetic disorders. Following the rapid advances in NGS technologies, the role of NGS in routine clinical practice will increase exponentially. Noninvasive prenatal diagnosis by using NGS is another application of this new technology. The most important step in the prenatal diagnostic procedures is obtaining fetal material to evaluate genetic condition. For years, invasive and noninvasive tests have been used to assess the fetal health, particularly chromosomal abnormalities, during the pregnancy. Noninvasive tests measure epiphenomena, which does not analyze the pathology underlying the clinical picture of interest. Their sensitivity and specificity have not also reached the expected level despite several studies. On the other hand, invasive tests have been found to be associated with significant risks for both the mother and fetus. The identification of cell-free fetal DNA in maternal circulation and analyzing this fetal material by using NGS opened up a new horizon in the field of reproductive medical care. Despite main advantages of NGS technology, the researchers and clinicians still have many concerns about the implementation of NGS in practice [52]. The interpretation of huge amount of data obtained by NGS technology, billing and insurance issues, duration and content of consent process, and disclosure of incidental findings and variants of unknown significance were the main challenges related to offering this technology [53].

Approximately 10 years ago, karyotyping was the gold standard in patients with intellectual disability but array-CGH analysis has become the first line diagnostic test replacing karyotyping and FISH nowadays. As evident from this example, approaches to the genetic-related diseases could change in parallel with the advances in technology and science. “Philadelphia chromosome” is one of the oldest evolutionary examples of personalized medicine by revealing the etiological factor and the treatment options of a disease step by step by using the genetic analysis as the improvements in the genetic techniques were established. In 1960, a small chromosome called Philadelphia chromosome was identified to be the cause of the chronic myeloid leukaemia (CML). It was shown in 1973 by the chromosome banding technique that this chromosome was a result of a translocation between chromosomes 9 and 22 [54, 55]. It took more than 10 years to identify the fusion gene* BCR/ABL *(breakpoint cluster region and v-abl Abelson murine leukaemia viral oncogene homologue) through the improvements of the molecular techniques [56]. The following studies revealed that this fusion gene resulted in activation of a tyrosine kinase, which led to the discovery of a tyrosine kinase inhibitor drug Gleevec that was shown to be a highly successful treatment for CML [57].

Genetic test that will be used in the diagnosis should be chosen very carefully, which might not be the newest or the most sophisticated one. Sometimes only a karyotype could be enough to identify the genetic condition in the patient instead of more complicated array-CGH or NGS methods. As the technology in genetics rapidly evolves, new insights in terms of data interpretation and genetic counseling including pretest counseling, return of results, and posttest counseling need to be considered. Databases and consortium reports regarding the experiences of the clinicians and geneticists are crucial for integration of genomics into clinical practice.

## 3. Conclusion

If the developments in genetics and computer technologies continue to progress at their current speed, history has shown us we can look forward to some amazing developments in human life in the very near future. Some realistic scenarios of human life in the future could even see us carrying identity cards, which include our genome characteristics, rather than the format we are currently using. Gene corrections, cloned individuals and organs, and even genetic-based techniques as a primary laboratory analysis in almost all human diseases for a clinician will no longer be a dream. We have come to the point nowadays where genetic testing is commercially available; the individual now has the possible means to access this delicate information named as direct to consumer (DTC) genetic testing. Contrary to the traditional hospital or physician based testing, accessing an individual's genetic information without medical or specialized interpretation has gradually been finding a place in our daily lives. Today, more than 25 companies, from all over the world, offer DTC service to the public. Serious concerns, however, regarding the use of this kind of service, have been raised in terms of misleading and incidental results derived from unproven or invalidated data. Moreover, there is also a significant risk for unauthorized use of sensitive genetic information by big business, particularly in the fields such as health insurance. On the other hand, DTC does provide early awareness of genetic diseases and thereby offers individuals the opportunity to play an active role in their own health care. The issue at stake here leads us to the same difficult medical ethics dilemma: patient autonomy and right to know one's genetic composition versus nonmaleficence.

To conclude, in parallel with the rapid developments in the field of genetic technologies, ethical and legal issues regarding the implementation of those technologies need to be addressed. Because use of personal genetic information looks certain to directly impact our daily lives in the near future, protocols need to be discussed in detail, with guidelines provided and updated regularly as part of a regulated multidisciplinary approach.
